# Epidemiological and clinical features of the emergency visits in a rural hospital in Cubal, Angola

**DOI:** 10.11604/pamj.2018.29.143.13780

**Published:** 2018-03-05

**Authors:** Miquel Turbau Valls, Eva Gil Olivas, Teresa López García, Domingas Piedade, Agostinho Pessela, Milagros Moreno Nicasio

**Affiliations:** 1 Emergency Department and Semicritical Area, Hospital de la Santa Creu i Sant Pau, Barcelona, Spain; 2 Autonomous University of Barcelona (UAB), Spain; 3 Hospital Nossa Senhora da Paz, Missão Catolica de Cubal, Benguela, Angola

**Keywords:** Tropical medicine, Angola, emergency service, malaria, low-and middle-income countries

## Abstract

**Introduction:**

There is scarce information on the profiles of patients attended in the Emergency Departments (ED) in rural Angola.

**Methods:**

Retrospective descriptive study including all the patients treated in the ED in Hospital Nossa Senhora da Paz (Cubal) during 6 months (December 2014- May 2015). The epidemiological and clinical data collected were: age, sex, shift, service assignment, reason for consultation and outcome (discharge, admission, referral or death).

**Results:**

A total of 2384 patients (53.4% women) were attended. The median age was 10 years (range: 0 - 96 years); 57.9% and 40.2% of them were under 17 and 5 years, respectively. No differences were observed regarding the assistance per shift, weekdays, weekends, or mean age per shift. The reason for consultation was registered in 69.9% of the patients; the most common were respiratory tract infections (20.5%), fever (14%), digestive diseases (13.6%) and malaria (10.4%). Up to 47.2% of the patients required in-hospital treatment and 1.3% were transferred to other hospitals. The patients admitted were significantly younger than the patients discharged (median age of 4 vs.16 years, p < 0.01). The mortality rate within the ED was 0.5%.

**Conclusion:**

Young patients were those who mostly required assistance in the ED. Infectious diseases were the most frequent reason for consultation. Pulmonary tuberculosis was suspected in one third of respiratory infections. The admission rate was high, especially in children under 5 years and in cases of malaria and malnutrition. Low referral rate and low mortality within the ED were observed.

## Introduction

Angola was stuck in a thirty-year civil war until 2002 after achieving its independence from Portugal in 1975. It has an estimated population of 25 million inhabitants, 48% of them under-15 years of age. Half of the population lives in non-urban areas, over one third is below the poverty threshold, and only one half has access to drinking water sources and waste treatment systems. The rate of physicians and nurses per 1000 inhabitants are 0.077 and 1.7, respectively. Life expectancy is currently about 51 years in men and 54 years in women. Despite a decreasing trend in the previous years, Angola is still one of the African countries with the highest maternal and under-5 year mortality rates [1-3]. Communicable diseases are the responsible of more than 50% of mortality in Angola. Measles, rabies, cholera, leprosy and yellow fever outbreaks are periodically reported [4]. This is considered an area with high transmission rate of Plasmodium falciparum malaria, with more than 2 million confirmed cases in 2014, although its related mortality has recently decreased [2,5]. Despite being considered underestimated, the prevalence of HIV infection among adults is 2.4%, with an antiretroviral treatment coverage of 42% of the population infected. Incidence of tuberculosis (TB) keeps increasing, with no conclusive data on multidrug-resistance related. The main causes of mortality are diarrheic diseases, respiratory infections and malaria. The main causes of child mortality are malaria (up to 33%), prematurity, asphyxia and neonatal tetanus. While mortality from caloric-protein malnutrition, respiratory infections, diarrheic syndromes and meningitis has decreased, it has increased mortality derived from complications of pre-term delivery and neonatal causes. It has also been reported an increase in other causes of mortality such as HIV, and cardiovascular causes (stroke or ischemic heart disease) [1,6].

The Hospital Nossa Senhora da Paz is in a peri-urban area of Cubal Sede, in Cubal municipality (province of Benguela) with an estimated population of 322000 inhabitants, where nearly half of them are under 15 years of age [7]. It has 294 beds (including 30 in the Malnutrition Unit and 150 in the TB Unit), an outpatient clinic, basic laboratory, and radiology and ultrasound service. The Emergency Department (ED) has four beds and is attended by a nurse with training in diagnosis and treatment on a 24-hour work shift with an on-call doctor if needed. Emergency care and treatment is accessible for all the population free of charge, whereas x-rays and lab tests are paid on an affordable tax by the patient. According to the hospital´s Annual Report, 17823 outpatients, 5098 ED visits and 657 natural deliveries were attended in 2014. Almost 5000 patients were admitted and 29 caesarean procedures were performed during this period. No specific emergency medical training or pre-hospital medical care services exist in most African countries, despite positive experiences that have been reported in Ghana and Tanzania [8,9]. A major pitfall is the great variability in infrastructure and human resources of EDs where many health centres are ran by personnel with basic medical training [10]. In consequence, there is scarce information in the literature focusing on pathologies and the clinical profiles of patients urgently attended in this geographical area. The present study aimed to describe the profile of pathologies and patients attended in the ED of a rural hospital in a low-income African country, outcome and mortality within the department. In addition, this study may give indirect information on the community health and resources that should be provided to health professionals.

## Methods

In this retrospective descriptive study we analysed all the patients treated in the ED of Hospital Nossa Senhora da Paz from December 2014 to May 2015. The information was obtained from the ED register where the nurse collects age, gender, shift (8am-3pm, 3pm-10pm, and 10pm-8am), destination (discharge, admission, referral or death), service assignment (internal medicine, paediatrics, orthopaedics, obstetrics) and reason for consultation (basic syndromic orientation based on clinical judgement). For further analysis, patients were grouped in age ranges: under 5 years, from 5 to 16 years, from 17 to 60 years and over 60 years. The reason for consultation was then grouped according to body systems and medical specialties. Permission for compilation of the data was obtained from the hospital direction office. All data were analysed with IBM-SPSS. Quantitative variables were expressed as mean ± standard deviation (SD) and median for non-normal distribution variables and qualitative variables as frequencies and percentages. Qualitative variables were compared using Chi-square test and quantitative variables using Student's T-test. Non-normal distribution variables were compared using non-parametric tests (U Mann-Whitney or Kruskall-Wallis). For analytical statistics, 95% Confidence Intervals (CI) were calculated, and a value of p < 0.05 was considered statistically significant.

## Results

**Figure 1:**
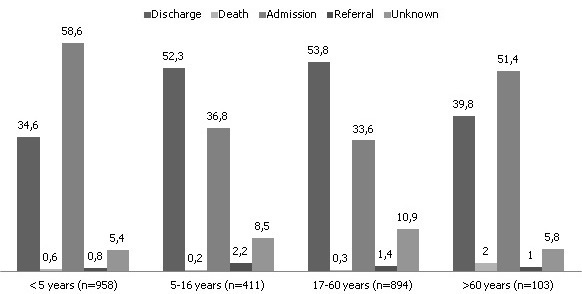
Destination according to age group

During the six months of the study 2384 patients were attended in the ED, with a daily mean (±SD) of 13 ± 5 patients, and a median of 13 (range 1-33). The mean number of visits per shift was 4.6, 4.0 and 4.4 in the morning, afternoon/evening and night, respectively. We observed a trend towards fewer visits in the afternoon (p=0.05) with no statistical differences in the attendance between weekdays and weekends (p=0.4). The median age of the patients was 10 years (range: 0-96) (women 16 years, men 6 years) whereas 53.4 % were female. Age and/or gender were not registered in 23 patients. There were no differences in the mean age per shift (p = 0.2). The distribution of patients according to the age range was: under 1 month: 21 (0.9%), 1-12 months: 239 (10%), 1-5 years: 698 (29.3%), 5-16 years: 411 (17.2%), 17-60 years: 894 (37.5%) and older than 60 years: 103 (4.3%). In overall, a 40.2% of patients were younger than 5 years, and a total of 57.9% were younger than 17 years. The distribution by service assignment was: Paediatrics (1308 patients; 55%), Internal Medicine (967 patients; 40.6%), Orthopaedics (62 patients; 2.6%) and Gynaecology/Obstetrics (41 patients; 1.7%). Reason for consultation according to the age group is shown in Table 1. In 701 patients (29.4%) no data was registered regarding the reason for consultation. Considering those patients with all data registered (69.6%), the most frequent syndromic diagnoses were respiratory infections (20.5%, CI 18.6-22.5), fever (14%, 95% CI 12.4-15.8), digestive syndromes (13.5%, 95% CI 12-15.3), malaria (10.4%, 95% CI 9-11.9), trauma/injuries and malnutrition. In case of malaria suspicion, a rapid diagnostic test was performed, and a thick blood smear and peripheral blood extension were performed later for confirmation. Table 2 shows the distribution of age groups for each syndromic diagnosis. Outcomes according to the age group are shown in Figure 1. A total of 1076 patients (45.1%) treated in the ED were discharged, 1126 (47.2%) required in-hospital treatment, 31 (1.3%) were referred to other centres and 12 (0.5%) died within the ED (data on the death causes were not available). The patients admitted (median age of 4 years) were usually younger than those discharged (median age of 16 years) (p < 0.0001) or transferred (median age of 15 years) (p = 0.02). Patients diagnosed of malnutrition had a median age of 1 year (range: 0-23 years), and had a higher rate of hospital admission (95.4% malnutrition vs. 44.9% other consultations, p < 0.001). Patients diagnosed of malaria had a median age of 5 years (range: 1-55 years), and also had a significantly higher rate of admission (77.9% malaria vs. 44.8% other consultations, p < 0.001).

**Table 1:** Reason for consultation to the Emergency Department according to age group

Age group	< 5 years n(%)	5-16 years n(%)	17-60 years n(%)	>60 years n(%)	Global n(%)
Respiratory infection ^a^	153(21.8)	33(11.7)	131(22.2)	23(27.1)	340(20.5)
Fever	126(18)	41(14.5)	62(10.5)	4(4.7)	233(14)
Digestive symptoms	120(17.1)	30(10.6)	64(10.8)	11(12.9)	225(13.5)
Malaria	81(11.6)	68(24)	23(4)	-	172(10.4)
Trauma and wounds	12(1.7)	48(17)	73(12.4)	4(4.7)	137(8.3)
Malnutrition	104(14.8)	3(1.1)	2(0.3)	-	109(6.6)
Anaemia	37(5.3)	11(3.9)	10(1.7)	1(1.2)	59(3.6)
Cardiovascular	2(0.3)	4(1.4)	67(11.4)	22(25.9)	95(5.7)
Skin and soft tissue	3(0.4)	1(0.3)	3(0.5)	-	7(0.4)
Gynaecology and Obstetrics	13(1.9)	-	15(2.5)	-	28(1.7)
Neurologic symptoms	8(1.1)	12(4.2)	32(5.4)	5(5.9)	57(3.4)
Nephro-urologic symptoms	4(0.6)	3(1.1)	22(3.7)	8(9.4)	37(2.2)
Others ^b^	38(5.4)	29(10.2)	86(14.6)	7(8.2)	161(9.7)
**Total**	**701(100)**	**283(100)**	**590(100)**	**85(100)**	**1659(100)**

a It includes 101 cases of suspected pulmonary tuberculosis(8 in <5 years, 6 in 5-16 years, 71 in 17-60 years and 16 in older than 60 years)

b Others: ophthalmology(5), otorhinolaryngology(36), intoxications/poisoning(8), psychiatry(4), tumours(20), metabolic disorders(13), human immunodeficiency virus(6), measles, dental pathology, sexual assault, pneumothorax

**Table 2:** Distribution of age groups within each diagnostic orientation

Age group	<5 years	5-16 years	17-60 years	>60 years	Total
Respiratory Infections	45%	9,7%	38,5%	6,8%	100%
Fever	54,1%	17,6%	26,6%	1,7%	100%
Digestive symptoms	53.3%	13.3%	28.4%	5%	100%
Malaria	47.1%	39.5%	13.4%	0%	100%
Trauma and wounds	8.7%	35%	53.3%	3%	100%
Malnutrition	95.4%	2.8%	1.8%	0%	100%
Anaemia	62.7%	18.6%	17%	1.7%	100%
Cardiovascular	2.1%	4.2%	70.5%	23.2%	100%
Skin and soft tissue	42.9%	14.2%	42.9%	0%	100%
Gyne/Obstetrics	46.4%	0%	53.6%	0%	100%
Neurologic symptoms	14%	21%	56.2%	8.8%	100%
Nephro-Urologic	10.8%	8.1%	59.5%	21.6%	100%
Others^*^	23.7%	18.1%	53.8%	4.4%	100%

* *Others* include: ophthalmology, otorhinolaryngology, intoxications/poisoning, psychiatry, tumours, metabolic disorders, sexual assault, human immunodeficiency virus, measles, dental pathology, pneumothorax

## Discussion

The population attended in the ED was very young, in concordance with the hospital´s reference population [7]. Children under five years of age accounted for 40% of the patients, in contrast with other studies in Tanzania and Ethiopia where this age range was four times smaller [8,11]. Consequently, half of the hospital admissions in 2014 were in Paediatrics department. This fact stresses the need for specific resources and paediatric training for the health professionals in the ED. While in Nigeria almost a third of the patients treated in the ED were over 60 years old, in Angola this age group accounted only for the 3.9% of the country's population, thus in our study, this older population represented only a small percentage [12]. Overall, infectious causes were the predominant reason for consultation. They accounted for over 40% of the cases including fever of unknown origin. Our data were congruent with other studies where respiratory infections, gastrointestinal syndromes and malaria were the most frequent reason to seek urgent medical aid in this area [1,6,13]. Respiratory infections in particular have been reported to be the second cause of mortality in the country [2]. In our study, pulmonary TB was suspected in 30% of the patients that consulted because of respiratory infections, mostly in adults. As a national reference centre for the diagnosis and treatment of TB, our hospital usually receives referrals from all over the country. In 2014, 470 new cases of pulmonary TB were diagnosed and 8% received second-line treatment for multidrug resistant TB (data from the Hospital Annual Report). In our study diarrheic syndromes were the third cause of consultation, especially in children. In Angola, these syndromes are the second most reported infection after malaria. This may be due to the high prevalence of intestinal parasitosis, reported in half of school-aged Angolan children, mainly ascariasis, giardiasis, hymenolepiasis and trichuriasis [2,14-16]. Specifically in the area of Cubal, intestinal parasitosis was reported to be present in 16% of children [15]. Respiratory and digestive syndromes, fever, malnutrition and malaria accounted for 83% of the consultations in children under 5 years, in agreement with previous reports highlighting these as the main causes of infant morbidity and mortality [1,6]. Suspicion of malaria motivated 15% of consultations in patients under 17 years old and a very high admission rate. A mortality rate related to malaria of 8.3% has been described in Cubal [17]. Despite the decrease in the incidence observed in this area, malaria still accounts for 35% of healthcare demand and 20% of hospital admissions [**2**]. Considering this high impact on the most vulnerable age groups, maintenance of the pharmacological supply and diagnostic tests represents a challenge and an urgent sanitary priority. The number of visits in the ED due to severe malnutrition was high. Most cases required in-hospital treatment, mainly children under 5 years of age. As previously reported, prevalence of acute and chronic malnutrition is 8.2 and 29.7%, respectively, and almost half of Angolan children under 5 years have stunted growth [1,18]. Considering these high rates, it is essential to promote nutrition protocols and strengthen specialised centres in its treatment. Gynaecological and obstetric consultations were fewer than reported in other studies, as these patients were usually attended (and registered) in the maternity centre instead [8,11]. According to data from the Hospital Annual Report 2014, these visits accounted for 28% of the emergencies attended. Angola is considered an endemic area of Schistosoma haematobium. A recent study reported a 61% prevalence of urinary schistosomiasis in children from Cubal, a higher rate than the national average 28% [15,16]. In our study visits due to urologic complaints were significantly higher in adults than in children. This may be due to the fact that younger patients were visited more frequently in the outpatient clinic while adult patients with more advanced clinical symptoms went directly to the ED.

A progressive increase in the incidence of chronic non-communicable diseases (NCD), classic cardiovascular risk factors and their related complications has been reported in most sub-Saharan Africa countries [3]. Ischemic heart disease is a frequent reason for consultation at ED [13]. More than a third of the Angolan population has hypertension, 8% has hyperglycaemia and 9% has a cardiovascular disease [1-3]. It has been reported a prevalence of peripheral arterial disease of 42.6% in this area [19]. Moreover, mortality attributable to these NCD has been estimated to be 24% in Angola [2,3]. In our study, the low rate of visits due to cardiovascular symptoms was similar to other series in the literature and it was related to the low mean age of the population attended [11]. However, when considering the group over 60 years, one every four patients consulted for cardiovascular related symptoms and the number of patients complaining of related neurological symptoms was also higher than average. These data revealed the growing relevance of these chronic NCD in the community and the need to implement screening, early detection and treatment protocols in the community health centres. Finally, the frequency of injuries was lower than in other studies in African countries where they have increased in recent years [2]. The lack of an orthopaedic surgeon in our hospital may be the cause of patients seeking medical assistance in the other municipal hospital with a permanent trauma service. Mortality within ED was low (0.5%). However, due to the short time spent by patients in the service prior to admission or discharge, a selection bias by failing to register some patients cannot be excluded. According to the Hospital Annual Report, the crude hospital mortality rate in 2014 was 7%, similar to other studies in comparable settings [12,20]. Regarding the destination of patients, our results supported those previously published in Ethiopia with admission and referral rates of 47% and 0.3% respectively [11]. The present study has several limitations. It was performed in a single centre. The reason for consultation was not recorded in almost one-third of the patients. In moments of great attendance, probably not all patients were registered by the nurse. Training and clinical capacity of the nursing staff were variable and limited. Together with the few available complementary explorations to support clinical suspicion, this fact may have contributed to the lack of accuracy in some syndromic orientation.

## Conclusion

The ED of our institution, located in a rural area of central Angola, attended a very young population. Infectious diseases were the most frequent causes for consultation, mainly respiratory infections, diarrheic syndromes and malaria. Approximately half of the patients required in-hospital treatment, especially in cases of malaria and malnutrition. The mortality and referral rates were low. Priority should be given to measures that guarantee permanently available treatment for malaria and specific paediatric and nutritional care programs for the healthcare providers.

### What is known about this topic

- There is great variability in infrastructure and human resources of Emergency Departments in African countries and scarce scientific information focusing on the pathologies and the clinical profiles of patients treated;
- Communicable diseases are the responsible of more than 50% of mortality in Angola, although non-communicable causes of mortality are rising;
- There is still a high incidence of malaria, acute and chronic malnutrition especially in Angolan children under 5 years of age.

### What this study adds

- In rural Angola, the Emergency Department attended predominantly a very young population with infectious diseases (mainly respiratory infections, diarrheic syndromes and malaria);
- High hospitalization rates were observed among children under 5 years, especially in cases of malaria and malnutrition;
- Pulmonary tuberculosis was suspected in one every three patients that consulted because of respiratory infections, mostly in adults.

## Competing interests

The authors declare no competing interest.

## References

[1] World Health Organization (WHO) Global Health Observatory Data Repository. Angola statistics summary (2002-present).

[2] Ministério da Saúde, República de Angola (2014). Plano Nacional de Desenvolvimento Sanitário 2012-2025.

[3] World Health Organization (WHO) (2014). Non-communicable Diseases (NCD) Country Profiles.

[4] Barrett Alan DT (2016). Yellow fever in Angola and beyond-The problem of vaccine supply and demand. N Engl J Med.

[5] World Health Organization (WHO) (2014). World Malaria Report..

[6] Simão R, Gallo PR (2013). Infant mortality in Cabinda, Angola: challenge to health public policies. Rev Bras Epidemiol.

[7] (2009). Administração Municipal do Cubal. Perfil do Município do Cubal, Provincia de Benguela.

[8] Reynolds TA, Mfinanga JA, Sawe HR, Runyon MS, Mwafongo V (2012). Emergency care capacity in Africa: a clinical and educational initiative in Tanzania. J Public Health Policy.

[9] Rockefeller AO, Donkor P (2014). The Ghana Emergency Medicine Collaborative. Acad Med.

[10] Molyneux E, Robertson A (2002). Emergency medicine in differently resourced settings: what can we offer each other?. Emerg Med J.

[11] Taye BW, Yassin MO, Kebede ZT (2014). Quality of emergency medical care in Gondar University Referral Hospital, Northwest Ethiopia: a survey of patients, perspectives. BMC Emerg Med.

[12] Desalu OO, Ojo OO, Busari OA, Fadeyi A (2011). Pattern of respiratory diseases seen among adults in an emergency room in a resource-poor nation health facility. Pan Afr Med J.

[13] Traoré A, Ouédraogo HZ, Sondo B, Guissou IP (2002). Medical emergencies in the Yalgado Ouedraogo national hospital of Ouagadougou: patients' profile and assessment of care practices. Sante.

[14] Oliveira D, Ferreira FS, Atouguia J, Fortes F, Guerra A, Centeno-Lima S (2015). Infection by intestinal parasites, stunting and anemia in school-aged children from southern Angola. PLoS One.

[15] Bocanegra C, Gallego S, Mendioroz J, Moreno M, Sulleiro E, Salvador F (2015). Epidemiology of schistosomiasis and usefulness of indirect diagnostic tests in school-age children in Cubal, central Angola. PLoS Negl Trop Dis.

[16] Sousa-Figueiredo JC, Gamboa D, Pedro JM, Fançony C, Langa AJ, Soares Magalhães RJ (2012). Epidemiology of malaria, schistosomiasis, geohelminths, anemia and malnutrition in the context of a demographic surveillance system in Northern Angola. PLoS ONE.

[17] Salvador F, Cossio Y, Riera M, Sánchez-Montalvá A, Bocanegra C, Mendioroz J (2015). Changes in malaria epidemiology in a rural area of Cubal, Angola. Malar J.

[18] UNICEF data. Nutritional status (Updated May 2016). Stunting disparities by residence and wealth quintile.

[19] Paquissi FC, Cuvinje AB (2016). Prevalence of peripheral arterial disease among adult patients attending outpatient clinic at a general hospital in South Angola. Scientifica.

[20] Ugare GU, Ndifon W, Bassey IAE, Oyo-Ita AE, Egba RN, Asuquo M (2012). Epidemiology of death in the emergency department of a tertiary health centre south-south of Nigeria. Afr Health Sci.

